# Xanthophyll-Rich Extracts from *Garcinia dulcis* Pulp as Potential Anti-Hepatocellular Carcinoma Functional Food

**DOI:** 10.3390/nu18040670

**Published:** 2026-02-18

**Authors:** Ulfa Kholili, Aji Bayu Wicaksono, Amal Arifi Hidayat, Ugroseno Yudho Bintoro, Soetjipto Soetjipto, Aryati Aryati, Muhammad Zulfikar Fiko Defianto, Muhammad Miftahussurur

**Affiliations:** 1 Doctoral Program of Medical Science, Faculty of Medicine, Universitas Airlangga, Surabaya 60132, Indonesia; ulfa-k@fk.unair.ac.id; 2 Division of Gastroentero-Hepatology, Department of Internal Medicine, Faculty of Medicine, Universitas Airlangga, Surabaya 60132, Indonesia; arifiamal@gmail.com; 3 Division of Gastroentero-Hepatology, Department of Internal Medicine, Dr. Soetomo General Academic Hospital, Surabaya 60286, Indonesia; 4 Internal Medicine Speciality Study Program, Department of Internal Medicine, Faculty of Medicine, Universitas Airlangga, Surabaya 60115, Indonesia; dr.ijauyab@gmail.com; 5 Helicobacter Pylori and Microbiota Study Group, Institute Tropical Disease, Universitas Airlangga, Surabaya 60132, Indonesia; 6 Division of Medical Oncology Hematology, Department of Internal Medicine, Faculty of Medicine, Universitas Airlangga, Surabaya 60132, Indonesia; ugroseno@fk.unair.ac.id; 7 Department of Medical Biochemistry, Faculty of Medicine, Universitas Airlangga, Surabaya 60132, Indonesia; soetjipto1950@gmail.com; 8 Department of Clinical Pathology, Faculty of Medicine, Universitas Airlangga, Surabaya 60132, Indonesia; aryati@fk.unair.ac.id; 9 Faculty of Medicine, Universitas Airlangga, Surabaya 60132, Indonesia; fikodefianto@gmail.com

**Keywords:** hepatocellular carcinoma, *Garcinia dulcis*, xanthophylls, network pharmacology, molecular docking, anticancer natural products

## Abstract

Introduction: Hepatocellular carcinoma (HCC) is the most common primary liver malignancy and remains a leading cause of cancer-related mortality worldwide. Despite recent advances in immunotherapy and targeted agents, treatment efficacy is frequently limited by tumor heterogeneity, drug resistance, and systemic toxicity. Natural products, particularly carotenoid-derived compounds, have emerged as promising multi-target anticancer agents. Xanthophylls, a class of oxygenated carotenoids, exhibit pleiotropic biological activities that are relevant to cancer therapy; however, their potential against HCC remains incompletely explored. This study aimed to systematically evaluate the anti-HCC potential of xanthophyll-rich extracts from *Garcinia dulcis* pulp using integrated metabolomic, in silico, and in vitro approaches. Methods: Xanthophyll-rich extracts from *G. dulcis* pulp were prepared using microwave-assisted extraction. Phytochemical profiling was performed using UHPLC–ESI–MS/MS. In silico analyses included bioactivity prediction, ADMET profiling, target identification, network pharmacology, pathway enrichment, and molecular docking against key HCC-related proteins (EGFR, BCL-2, and mTOR). In vitro antiproliferative activity was assessed using MTT assays on HepG2 and Huh7 hepatocellular carcinoma cell lines, with THLE-2 normal hepatocytes used as controls. Results: Metabolomic analysis revealed a xanthophyll-dominated profile, with zeaxanthin and lutein as the major constituents, alongside fucoxanthin, astaxanthin, β-cryptoxanthin, β-carotene, and canthaxanthin. In silico predictions demonstrated high antineoplastic and pro-apoptotic activities, with strong involvement in the HIF-1, EGFR, PD-1/PD-L1, JAK–STAT, and mTOR signaling pathways. Molecular docking confirmed stable and high-affinity interactions of xanthophylls with EGFR, BCL-2, and mTOR. In vitro assays showed selective cytotoxicity against HCC cells, with IC_50_ values of 42.8 ± 3.6 µg/mL (HepG2) and 58.4 ± 4.9 µg/mL (Huh7), while exhibiting significantly lower toxicity toward normal hepatocytes. Conclusions: Xanthophyll-rich extracts from *Garcinia dulcis* pulp exhibit potent and selective anti-hepatocellular carcinoma activity through multi-target mechanisms involving oncogenic signaling, apoptosis regulation, and tumor metabolism. These findings support the translational potential of *G. dulcis* xanthophylls as promising natural candidates for further development in HCC therapy.

## 1. Introduction

Hepatocellular carcinoma (HCC) is the most prevalent form of primary liver cancer and represents a major global health burden [1]. According to the Global Cancer Observatory, liver cancer accounted for approximately 866,000 new cases and 759,000 deaths worldwide in 2022, ranking it among the top three causes of cancer-related mortality globally [2]. The lethality of HCC is driven by multiple factors, including late-stage diagnosis, tumor heterogeneity, and the frequent presence of cirrhosis or chronic hepatitis, which severely restricts treatment options and tolerance [3]. Even with advances in imaging and surveillance, many patients still present with unresectable disease, emphasizing the urgent need for novel therapeutic strategies that are both effective and safe for patients with compromised liver function [4].

In recent years, systemic therapy for advanced HCC has evolved rapidly, particularly with the introduction of immune checkpoint inhibitors and anti-angiogenic agents [5]. The combination of atezolizumab and bevacizumab is currently recommended as a first-line therapy due to improved overall and progression-free survival compared with sorafenib [6]. However, the clinical benefit of these therapies is limited by primary and acquired resistance, immune-related adverse events, and bleeding risk associated with VEGF inhibition [7]. Moreover, the molecular heterogeneity of HCC leads to variable responses across patients, highlighting the need for multi-target agents capable of modulating several oncogenic and inflammatory pathways simultaneously, preferably with lower systemic toxicity.

Natural products remain one of the richest sources of anticancer agents, particularly compounds capable of acting on multiple molecular targets [8]. Among these, xanthophylls, a class of oxygenated carotenoids, have gained increasing attention due to their ability to regulate key hallmarks of cancer, including proliferation, apoptosis, oxidative stress, angiogenesis, and inflammation [9]. Unlike simple antioxidants, xanthophylls can exert context dependent pro oxidant effects in cancer cells, leading to mitochondrial dysfunction, caspase activation, and tumor-selective apoptosis [10]. In liver cancer models, carotenoids such as astaxanthin have been shown to suppress HCC cell growth through inhibition of oncogenic pathways, including NF-κB, Wnt/β-catenin, and MAPK signaling, while also enhancing apoptotic responses [11]. These properties make xanthophylls highly attractive candidates for multi-pathway targeting in HCC.

*Garcinia dulcis* (mundu), a tropical fruit native to Southeast Asia, has long been used in traditional medicine and is increasingly recognized as a source of bioactive phytochemicals [12]. Experimental studies have demonstrated that extracts from *G. dulcis* fruit exhibit cytotoxic and apoptosis-inducing effects in HepG2 hepatocellular carcinoma cells, supporting its relevance as a source of anti-HCC compounds [13]. While most prior investigations of the *Garcinia* genus have focused on xanthones and flavonoids, the xanthophyll-rich fraction of *G. dulcis* pulp remains largely unexplored, despite the strong mechanistic rationale for carotenoid-mediated anticancer activity [14]. Therefore, this study integrates in silico target prediction and molecular docking with in vitro HCC cell-based assays to systematically evaluate the anti-hepatocellular carcinoma potential of xanthophyll-rich extracts from *G. dulcis* pulp, aiming to identify mechanistically supported, multi-target natural candidates for future HCC drug development. Unlike previous studies that mainly focused on isolated carotenoids or marine-derived xanthophylls, this study is the first to systematically characterize a xanthophyll-rich extract from the edible pulp of *Garcinia dulcis* and to integrate metabolomic profiling with network pharmacology and experimental validation specifically in hepatocellular carcinoma models, thereby providing a plant-based, food-relevant perspective for multi-target HCC intervention.

## 2. Materials and Methods

### 2.1. Preparation of Samples and Extraction Procedure

Fresh fruits of *Garcinia dulcis* were obtained from a commercial fruit market in Yogyakarta, Indonesia, and taxonomically authenticated (voucher specimen No. 055/LSB/REP/VI/2025). The pulp fraction was separated, thoroughly washed with ultrapure water generated by a Milli-Q IQ 7003 system (MilliporeSigma, Burlington, MA, USA), and evenly distributed on stainless-steel trays in a thin layer (5 mm). Drying was carried out in a Memmert U universal hot-air oven (Memmert GmbH, Schwabach, Germany) at 40 °C until the residual moisture content reached approximately 10% (*w*/*w*), a level chosen to minimize xanthophyll degradation [15]. The dried material was subsequently ground to a fine powder (0.5 mm particle size) using a GRINDOMIX GM 200 knife mill (Retsch GmbH, Haan, Germany), whose high-power motor and stainless-steel blades enabled rapid homogenization with minimal thermal stress, thereby preserving thermolabile pigments.

Microwave-assisted extraction (GDP-MAE) was performed using a MARS 6 microwave reactor (CEM Corporation, Matthews, NC, USA) fitted with PTFE vessels and One-Touch™ temperature-controlled programming [16,17]. One gram of the powdered pulp was suspended in 15 mL of an ethanol–water mixture (60:40, *v*/*v*) and transferred into 40 mL sealed PTFE vessels. Extraction was conducted at 600 W using intermittent irradiation cycles (30 s on/30 s off) to maintain the internal temperature at 50 °C for 5 min, thereby enhancing solvent penetration and xanthophyll solubilization while avoiding localized overheating. After cooling, the extracts were filtered through Whatman No. 1 filter paper (Whatman, Maidstone, UK) and concentrated under reduced pressure at 40 °C using a Büchi rotary evaporator (Büchi Labortechnik AG, Flawil, Switzerland). This extraction workflow integrates environmentally friendly solvents with controlled microwave technologies, enabling efficient and reproducible recovery of lutein, zeaxanthin, and structurally related xanthophylls from *Garcinia dulcis* pulp waste while preserving their chemical integrity.

### 2.2. Metabolomic Profiling of Garcinia dulcis Pulp Extracts via UHPLC-ESI-MS/MS

Prior to chromatographic analysis, *Garcinia dulcis* pulp extracts obtained by microwave-assisted extraction (GDP-MAE) were diluted in a methanol–dichloromethane mixture (1:1, *v*/*v*; LC–MS grade methanol and dichloromethane, Merck KGaA, Darmstadt, Germany) and passed through a 0.22 µm nylon membrane syringe filter (MilliporeSigma, Burlington, MA, USA) to remove particulate contaminants. The clarified solutions were adjusted to a final concentration of 1 mg/mL before injection into the UHPLC system. Reference standards were prepared following the protocol described by Balasubramaniam et al. (2020), with minor modifications [18]. Briefly, individual standards (purity ≥ 99.5%, Sigma-Aldrich^®^, St. Louis, MO, USA) were dissolved in a 50:50 (*v*/*v*) methanol–dichloromethane solvent to produce stock solutions of 10 mg/mL, which were stored at −20 °C under light-protected conditions until analysis.

Chemical profiling was performed using a high-resolution UHPLC-ESI-MS/MS platform consisting of a Dionex Ultimate 3000 UHPLC coupled to a Q Exactive Hybrid Quadrupole-Orbitrap mass spectrometer (Thermo Fisher Scientific, Waltham, MA, USA). Chromatographic separation was achieved on a reversed-phase Acquity UPLC BEH C18 column (50 mm × 2.1 mm, 1.7 µm; Waters Corporation, Milford, MA, USA). The autosampler temperature was maintained at 4 °C, while the column oven was set to 35 °C, and 3 µL of each sample was injected. Elution was carried out at a flow rate of 0.3 mL/min using a binary solvent system composed of 0.1% formic acid in water (mobile phase A) and 0.1% formic acid in acetonitrile (mobile phase B; formic acid and acetonitrile, Merck KGaA, Darmstadt, Germany). The gradient started at 5% B, increased linearly to 99% between 2 and 20 min, was held at 99% until 25 min, and then returned to initial conditions by 30 min.

Mass spectrometric detection was conducted in both positive and negative electrospray ionization modes over an *m*/*z* range of 100–1000 with a resolving power of 140,000 FWHM. The operating parameters included a sheath gas flow rate of 35, auxiliary gas flow of 12, capillary temperature of 320 °C, spray voltage of 3.7 kV, and an S-lens RF level of 55 V. Tandem MS spectra were acquired using a normalized collision energy of 35 eV. Data acquisition and processing were performed using Xcalibur software (version 2.1.0; Thermo Fisher Scientific, Waltham, MA, USA).

### 2.3. In Silico Analyses and Computational Workflow

#### 2.3.1. Bioactivity Prediction and Pharmacokinetic Profiling

The therapeutic potential of phytochemicals identified from *Garcinia dulcis* pulp (GDP) extract against hepatocellular carcinoma was evaluated using the PASS (Prediction of Activity Spectra for Substances) platform provided by WAY2DRUG (https://way2drug.com/PassOnline/ (accessed on 17 December 2025)) [19]. This web-based tool employs a structure–activity relationship (SAR) algorithm that compares submitted molecular structures with a curated library of biologically characterized compounds to estimate probable pharmacological activities. Compounds exhibiting a probability of activity (Pa) greater than 0.7 were classified as strong candidates, while a Pa threshold of 0.4 was applied for preliminary screening [20]. Higher Pa values indicate greater predictive reliability. To further assess pharmaceutical suitability, pharmacokinetic behavior, toxicity risk, and drug-likeness were analyzed using ADMET descriptors and Lipinski’s Rule of Five [21,22]. Molecular features were derived from canonical SMILES notations and processed using ADMETLab 3.0 and ProTox-III platforms, with compound information obtained from the PubChem database [21,23,24].

#### 2.3.2. Protein Target Identification and Analysis

Putative protein targets of GDP extract-derived compounds were predicted using the SuperPred server (https://prediction.charite.de/ (accessed on 17 December 2025)) [25]. Canonical SMILES representations of each compound were submitted, and only targets with prediction confidence scores of at least 80% (on a 0–100% scale) were retained for further analysis. Genes and proteins implicated in hepatocellular carcinoma were retrieved from the GeneCards database (https://www.genecards.org/ (accessed on 17 December 2025)) [26]. Overlapping targets between GDP-derived compounds and HCC-related genes were identified using Venn diagram analysis generated via the Bioinformatics web tool (https://bioinformatics.psb.ugent.be/webtools/Venn/ (accessed on 17 December 2025)). These shared targets were subsequently selected for protein–protein interaction network construction.

#### 2.3.3. Network Pharmacology Analysis

Protein–protein interactions among the overlapping targets were investigated using the STRING database (version 12.0; https://string-db.org/ (accessed on 17 December 2025)), which integrates experimentally validated and computationally predicted associations based on physical binding and functional linkage [27]. The analysis was limited to Homo sapiens, and all receptor-related interactions were included. A stringent confidence score threshold of 0.9 was applied to ensure high-reliability interactions [28]. The resulting interaction network was exported in TSV format and imported into Cytoscape (version 3.10.1; https://cytoscape.org/ (accessed on 17 December 2025)) for visualization and topological analysis. Key network metrics, including degree centrality, closeness centrality, and betweenness centrality, were calculated to identify hub proteins and functionally critical nodes within the network [29].

#### 2.3.4. Gene Ontology and Pathway Enrichment Analysis

Biological interpretation of the predicted target genes was carried out using pathway and functional annotation based on the Kyoto Encyclopedia of Genes and Genomes (KEGG) and Gene Ontology (GO) resources. KEGG pathway reconstruction was performed using Python (version 3.11) in combination with the Bioservices package (version 1.11.2), which enables automated querying of the KEGG database (https://www.genome.jp/kegg/pathway.html, accessed on 17 December 2025) [30,31]. Compound names and molecular formulas were first matched to KEGG compound identifiers and subsequently linked to their annotated metabolic and signaling pathways.

Gene Ontology analysis was conducted to categorize GDP extract-associated targets into the three principal domains, Biological Process, Cellular Component, and Molecular Function, using curated annotation datasets [32]. Statistical enrichment was evaluated using a hypergeometric distribution to identify GO terms and KEGG pathways that were significantly over-represented compared with the background gene universe [33]. To control for multiple hypothesis testing, *p*-values were adjusted using the Benjamini–Hochberg false discovery rate (FDR) method, and terms with FDR-corrected values below 0.05 were considered statistically significant. Enrichment strength was expressed as the ratio between observed and expected gene counts [34].

#### 2.3.5. Molecular Docking and Binding Interaction Analysis

Protein ligand docking simulations were conducted using CB-Dock2, a blind docking platform guided by automated cavity detection (https://cadd.labshare.cn/cb-dock2/index.php (accessed on 17 December 2025)) [35]. This system integrates binding-site recognition, docking, and homologous structure fitting to improve both docking precision and computational efficiency. The software identifies potential ligand binding cavities, automatically defines docking boxes, and ranks binding poses based on AutoDock Vina (version 1.2.0) affinity scores [36]. Three-dimensional interaction patterns were visualized to examine ligand receptor binding modes. To further refine pocket prediction, the CurPocket curvature-based cavity detection algorithm was applied. Three-dimensional structures of GDP extract-derived ligands were retrieved from PubChem, whereas target protein structures, including EGFR (PDB ID: 1M17), BCL2 (PDB ID: 2W3L), and MTOR (PDB ID: 4JSV), were downloaded from the RCSB Protein Data Bank (https://www.rcsb.org (accessed on 17 December 2025)). All crystallographic water molecules were automatically removed by the CB-Dock2 server prior to docking to avoid interference with ligand binding. Although EGFR (PDB ID: 1M17) and BCL-2 (PDB ID: 2W3L) are available as ligand-bound complexes, blind docking was intentionally employed to maintain methodological consistency across all targets and to avoid bias toward predefined binding pockets. This approach allows unbiased exploration of potential interaction sites, particularly for large and flexible ligands such as xanthophylls, which may adopt alternative binding modes. The predicted cavities were subsequently compared with known active sites to ensure biological plausibility of the docking poses. Because blind docking was employed, ligands were not constrained to predefined crystallographic binding sites, and therefore occupancy of identical canonical pockets across all ligands was not enforced. Instead, binding poses were ranked based on affinity within automatically detected cavities, which were subsequently evaluated for consistency with known functional regions of each target protein.

### 2.4. In Vitro Anticancer Activity Assay

The antiproliferative activity of *Garcinia dulcis* pulp extracts prepared by microwave-assisted extraction (GDP-MAE) was assessed against hepatocellular carcinoma using human liver cancer cell lines HepG2 and Huh7, along with normal human hepatic epithelial cells (THLE-2). All cell lines were obtained from the American Type Culture Collection (ATCC, Manassas, VA, USA). Cells were exposed to GDP extracts at concentrations ranging from 0 to 500 µg/mL. Stock solutions of *Garcinia dulcis* extracts and sorafenib were prepared in dimethyl sulfoxide (DMSO; Sigma-Aldrich, St. Louis, MO, USA) and diluted in culture medium such that the final solvent concentration did not exceed 0.2% (*v*/*v*) in any treatment condition. Equivalent volumes of DMSO were added to untreated control wells, and preliminary solvent control experiments confirmed that DMSO at this concentration had no significant effect on cell viability. Cell viability was determined using the MTT colorimetric assay (Thermo Fisher Scientific, Waltham, MA, USA). Cells were seeded into 96-well plates and allowed to adhere for 24 h at 37 °C in a humidified incubator with 5% CO_2_. Following attachment, cells were treated with various concentrations of GDP extracts for 48 h. Subsequently, 20 µL of MTT reagent (5 mg/mL) was added to each well and incubated for 4 h to allow formazan formation. The resulting crystals were solubilized with 100 µL of dimethyl sulfoxide (DMSO; Sigma-Aldrich, St. Louis, MO, USA), and absorbance was recorded at 570 nm using a microplate reader. Sorafenib (Sigma-Aldrich, St. Louis, MO, USA) served as the positive control. Cell viability was expressed as a percentage of untreated controls, and IC_50_ values were calculated using nonlinear regression analysis. All experiments were performed in triplicate in three independent experiments, and data are reported as mean ± standard deviation [37].

### 2.5. Data Processing and Statistical Analysis

All statistical evaluations were carried out using GraphPad Prism Version 10.6.1 Premium for macOS (GraphPad Software, Boston, MA, USA). Experimental data are presented as mean ± standard deviation (SD). Half-maximal inhibitory concentration (IC_50_) values were obtained by nonlinear curve fitting using a variable-slope regression model. The distribution of the data was examined using the Shapiro–Wilk normality test. Each in vitro experiment was performed in triplicate (*n* = 3). For datasets showing normal distribution, differences among groups were analyzed using one-way or two-way analysis of variance (ANOVA), as appropriate. Statistical significance was defined at *p* < 0.05 with a confidence level of 95%.

## 3. Results

### 3.1. Metabolomic Profiling of GDP Extract Compounds

UHPLC-HRMS analysis of the microwave-assisted *Garcinia dulcis* pulp extract (GDP-MAE) revealed a xanthophyll-dominated profile with marked quantitative differences among carotenoids (Table 1). Zeaxanthin (29.8 ± 2.1 mg/100 g) and lutein (21.6 ± 1.8 mg/100 g) were the predominant constituents, eluting at 10.187 ± 0.051 and 10.476 ± 0.054 min, respectively, indicating that the GDP extract is particularly enriched in polar xanthophylls. β-Cryptoxanthin was present at intermediate levels (6.1 ± 0.5 mg/100 g; 3.310 ± 0.010 min), whereas β-carotene (3.4 ± 0.3 mg/100 g; 19.900 ± 0.200 min) represented the major non-oxygenated carotenoid. In contrast, more structurally complex marine type xanthophylls were detected at lower abundance, including fucoxanthin (1.9 ± 0.2 mg/100 g; 11.042 ± 0.020 min), astaxanthin (1.3 ± 0.1 mg/100 g; 11.810 ± 0.050 min), and canthaxanthin (1.2 ± 0.1 mg/100 g; 3.110 ± 0.020 min).

### 3.2. In Silico Analysis

In silico profiling revealed that *Garcinia dulcis* xanthophylls exhibit strong predicted anticancer activity against hepatocellular carcinoma (Table 2). All compounds showed high antineoplastic and pro-apoptotic activities (Pa = 0.900–0.973) together with robust TP53 expression–enhancing potential (Pa = 0.731–0.934), indicating multi-mechanistic anticancer relevance. Fucoxanthin demonstrated the highest antineoplastic probability (Pa = 0.973) with moderate predicted toxicity (LD_50_ = 130 mg/kg; class 3), whereas astaxanthin and β-carotene showed lower toxicity (LD_50_ = 1190–1510 mg/kg; class 4). In contrast, zeaxanthin, lutein, and β-cryptoxanthin were predicted to be highly bioactive but more acutely toxic (LD_50_: 10 mg/kg; class 2). Drug-likeness analysis indicated that only fucoxanthin, astaxanthin, and lutein satisfied Lipinski’s criteria, although most xanthophylls failed Pfizer and GSK filters, reflecting their highly lipophilic carotenoid structures. Although lutein is widely used in ophthalmic supplementation, its rejection by Pfizer and GSK rules reflects unfavorable oral drug-like properties (e.g., high lipophilicity, low aqueous solubility, and limited systemic bioavailability), rather than lack of biological activity. We have previously mentioned this in the manuscript.

**Figure 1:**
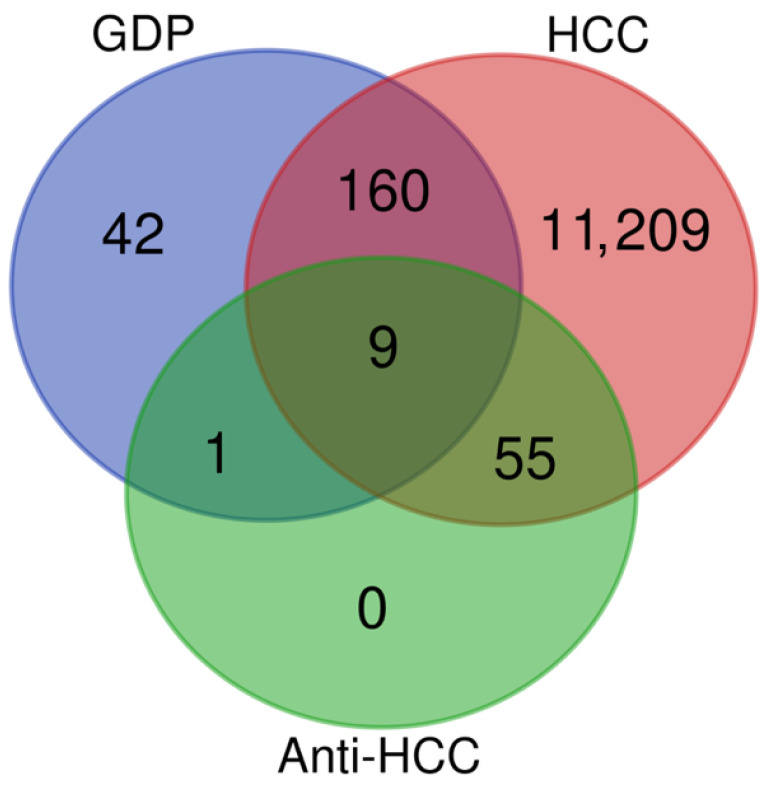
Venn Diagram of Overlapping Targets Between *Garcinia dulcis* Pulp Compounds, Hepatocellular Carcinoma-Associated Genes, and Anti-HCC Targets.

Venn diagram analysis demonstrated a focused intersection between *Garcinia dulcis* pulp (GDP) extract-derived targets, hepatocellular carcinoma (HCC)-associated genes, and established anti-HCC targets (Figure 1). Of the 202 predicted GDP-related targets, 169 overlapped with HCC-associated genes, indicating a strong disease relevance of GDP phytochemicals. Importantly, nine targets were shared across all three datasets (GDP, HCC, and anti-HCC), identifying a core set of proteins most likely to mediate the therapeutic effects of GDP against HCC. In contrast, a large number of genes were exclusive to HCC (11,209), reflecting the complexity of HCC pathobiology, while only minimal overlap was observed between GDP and anti-HCC targets alone.

**Figure 2:**
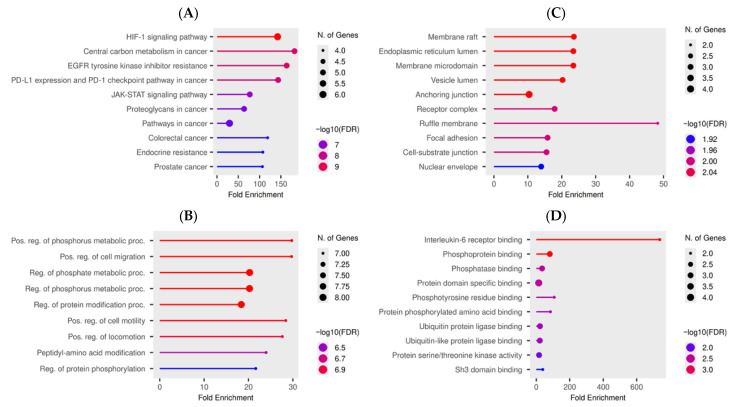
Functional and pathway enrichment analysis of core anti-hepatocellular carcinoma targets derived from *Garcinia dulcis* xanthophylls: (**A**) KEGG pathway enrichment showing major signaling pathways involved in HCC progression and therapeutic resistance; (**B**) Gene Ontology (GO) biological processes highlighting regulation of cell migration, phosphorylation, and signal transduction; (**C**) GO cellular components indicating localization in membrane-associated and receptor complexes; (**D**) GO molecular functions illustrating kinase activity, cytokine receptor binding, and protein–protein interaction functions. Bubble size represents gene count, and color intensity reflects statistical significance.

Functional enrichment analysis of the nine core *Garcinia dulcis*-derived anti-HCC targets revealed significant involvement in key oncogenic and tumor-progression pathways (Figure 2). KEGG analysis showed strong enrichment in HIF-1, EGFR tyrosine kinase inhibitor resistance, PD-1/PD-L1 checkpoint signaling, JAK-STAT, and proteoglycan pathways, all of which are central regulators of hepatocellular carcinoma growth, immune evasion, and therapeutic resistance (Figure 2A). Gene Ontology biological process terms were dominated by positive regulation of cell migration, motility, phosphorylation, and protein modification, indicating roles in invasion and signal transduction (Figure 2B). Cellular component analysis localized these targets mainly to membrane rafts, receptor complexes, focal adhesions, and vesicular compartments, consistent with active signaling hubs in cancer cells (Figure 2C). Molecular function enrichment further highlighted interleukin-6 receptor binding, phosphotyrosine and serine/threonine kinase activity, ubiquitin ligase binding, and SH3 domain interactions, underscoring the involvement of inflammatory, oncogenic, and protein-turnover pathways in GDP-mediated anti-HCC effects (Figure 2D).

**Figure 3:**
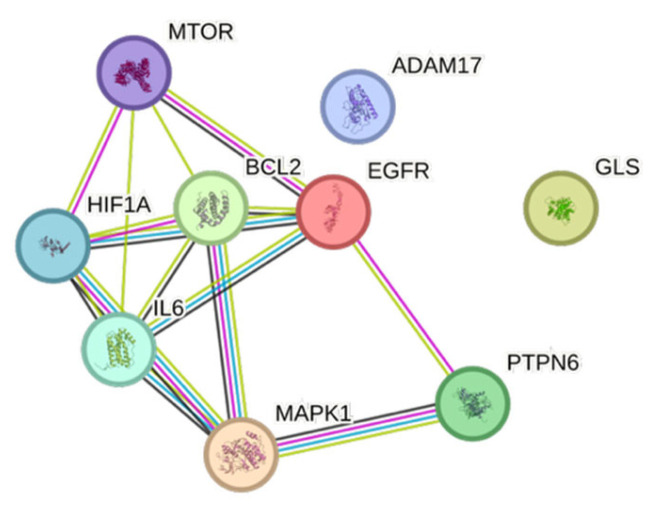
Protein–protein interaction (PPI) network of core anti-hepatocellular carcinoma targets predicted for *Garcinia dulcis* xanthophylls. Hub proteins (e.g., EGFR, BCL2, MAPK1, IL6, and HIF-1A) are central regulators of apoptosis, growth factor signaling, inflammation, and hypoxia-related pathways in HCC.

Protein–protein interaction (PPI) analysis of the nine core anti-hepatocellular carcinoma targets revealed a tightly connected signaling network centered on key oncogenic and survival regulators (Figure 3). BCL2, EGFR, MAPK1, IL6, and HIF-1A formed the main interaction hub, showing extensive cross-talk between apoptosis control, growth factor signaling, inflammatory pathways, and hypoxia responses. mTOR and ADAM17 were functionally linked to this core module, supporting roles in metabolic reprogramming and receptor shedding, while PTPN6 provided regulatory feedback on kinase signaling. In contrast, GLS appeared more peripherally connected, suggesting a supportive role in metabolic adaptation rather than direct oncogenic signaling.

Molecular docking revealed that *Garcinia dulcis* xanthophylls interact strongly with the key hepatocellular carcinoma targets EGFR, BCL-2, and mTOR, indicating a robust multi-target binding profile (Table 3). All compounds exhibited high affinity for EGFR (−8.6 to −8.9 kcal/mol) and BCL-2 (−8.8 to −9.0 kcal/mol), values that are close to the reference drug doxorubicin (−9.8 and −9.1 kcal/mol, respectively), supporting their potential to interfere with growth factor signaling and anti-apoptotic mechanisms. For mTOR, which is a central regulator of tumor metabolism and survival, fucoxanthin showed the strongest binding among GDP compounds (−10.5 kcal/mol), followed by astaxanthin (−9.9 kcal/mol), β-carotene (−9.7 kcal/mol), and canthaxanthin (−9.6 kcal/mol), approaching the affinity of doxorubicin (−10.9 kcal/mol). However, it is noteworthy that docking results are predictive and not a substitute for binding kinetics.

Molecular docking analysis demonstrated that all investigated xanthophylls exhibited stable binding conformations within energetically favorable cavities of EGFR, BCL-2, and mTOR. Despite structural differences among the ligands, the predicted binding modes were characterized by consistent engagement of hydrophobic regions within the target proteins, reflecting the highly lipophilic polyene backbone of xanthophylls. For EGFR, ligand binding occurred predominantly within surface-accessible cavities adjacent to the kinase domain, suggesting potential interference with receptor signaling rather than direct ATP-competitive inhibition. In the case of BCL-2, xanthophylls occupied hydrophobic grooves associated with anti-apoptotic regulation, supporting a mechanism involving disruption of survival signaling. Docking to mTOR revealed deep cavity engagement, consistent with modulation of metabolic and growth-related signaling pathways. Overall, the binding patterns were comparable across xanthophylls and the reference ligand doxorubicin, as reflected by their similar affinity scores.

### 3.3. In Vitro Analysis

The *Garcinia dulcis* pulp extract exhibited marked and selective cytotoxicity toward hepatocellular carcinoma cells compared with normal hepatocytes (Table 4). The extract inhibited HepG2 and Huh7 cell proliferation with IC_50_ values of 42.8 ± 3.6 µg/mL and 58.4 ± 4.9 µg/mL, respectively, whereas substantially lower toxicity was observed in normal THLE-2 cells (IC_50_ = 182.6 ± 15.3 µg/mL). This translated into favourable selectivity indices of 4.27 for HepG2 and 3.13 for Huh7, indicating preferential targeting of malignant cells. By comparison, the reference drug sorafenib demonstrated greater cytotoxic potency against HepG2 and Huh7 (IC_50_ = 6.2–7.9 µM) but also significantly higher toxicity toward THLE-2 cells (IC_50_ = 14.8 ± 1.4 µM), resulting in lower selectivity indices (1.87–2.39). One-way ANOVA with Tukey’s post hoc test confirmed significant differences between cancer and normal cells (*p* < 0.0001).

## 4. Discussion

The present study demonstrates that *Garcinia dulcis* pulp harbors a xanthophyll-rich phytochemical profile, with zeaxanthin and lutein as the predominant carotenoids, alongside lesser amounts of fucoxanthin, astaxanthin, β-cryptoxanthin, β-carotene, and canthaxanthin. This aligns with broader evidence that dietary carotenoids act as bioactive compounds with antioxidant and anticancer properties across multiple cancer models [38]. Carotenoids have been shown to modulate several cancer hallmarks, including cell proliferation, apoptosis resistance, angiogenesis, invasion, and metastatic potential, supporting their relevance for HCC research [39,40]. The differential abundance of these compounds in *G. dulcis* suggests that polar xanthophylls, in particular, may contribute to the extract’s biological activity due to their distinct chemical and functional properties. Although only nine overlapping targets were identified, this does not imply limited biological relevance, as these proteins represent central hubs within highly interconnected oncogenic networks. Importantly, target mapping indicated that multiple xanthophylls (particularly fucoxanthin, astaxanthin, lutein, and zeaxanthin) converge on several of these proteins, suggesting a cooperative multi-compound effect rather than dominance by a single molecule. This pattern supports a systems-level mechanism in which the extract acts through polypharmacology, consistent with the multi-target nature of functional food–derived bioactives.

In silico profiling provided evidence that GDP xanthophylls possess high predicted antineoplastic and apoptosis-enhancing probabilities, with significant potential to modulate TP53 expression and related oncogenic pathways. Such multi-mechanistic anticancer potential is consistent with the established understanding that carotenoids can exert anti-proliferative effects through induction of cell cycle arrest and apoptosis and regulation of growth factor signaling [41]. For example, fucoxanthin has been reported to inhibit cancer cell proliferation and induce apoptosis across a range of cancer types by modulating PI3K/Akt and other downstream survival pathways [42]. These mechanisms underline the capacity of xanthophylls to engage multiple intracellular signaling cascades that are dysregulated in HCC.

The integration of compound target associations with disease and anti-HCC targets revealed a specific subset of nine shared proteins that likely mediate the biological effects of GDP extract constituents. Functional enrichment highlighted involvement in hypoxia-related HIF-1 signaling, EGFR tyrosine kinase inhibitor resistance, PD-1/PD-L1 checkpoint regulation, and JAK-STAT signaling, pathways that are critically implicated in HCC progression and therapeutic resistance. This is supported by literature demonstrating that carotenoids can influence key nodes of oncogenic and survival networks, including modulation of transcription factors and signaling kinases relevant to apoptosis, oxidative stress responses, and immune modulation [38,43]. Thus, the mechanistic relevance of GDP targets situates these xanthophylls within a biologically plausible anticancer framework.

Molecular docking further corroborated that *G. dulcis* xanthophylls bind effectively to pivotal HCC regulators such as EGFR, BCL-2, and mTOR, suggesting potential interference with growth factor signaling, apoptotic inhibition, and metabolic adaptation in liver cancer cells. Such polypharmacological binding patterns are consistent with observations in other natural carotenoids, such as astaxanthin, which has been shown to exert anticancer effects through downregulation of anti-apoptotic proteins like BCL-2, upregulation of pro-apoptotic pathways, and modulation of inflammation-associated transcription factors like NF-κB and STAT3 [44,45]. These mechanistic attributes support the utility of xanthophyll-enriched extracts as potential multi-target agents in HCC management. It should be noted that the docking analysis was designed to support target relevance and multi-target engagement rather than to establish definitive binding-site equivalence; therefore, the observed interactions are interpreted at the level of functional plausibility and comparative affinity rather than precise residue-level binding specificity.

Importantly, the in vitro data demonstrated that the *G. dulcis* extract exhibits selective cytotoxicity toward HCC cell lines while sparing normal hepatocytes, yielding higher selectivity indices than the current standard therapy sorafenib. This selective profile suggests that GDP xanthophylls may preferentially target malignant cells, likely through the integrated mechanisms described above, including induction of apoptosis, disruption of survival signaling, and potential modulation of oxidative stress. The comparison with sorafenib should be interpreted cautiously, as the extract is a complex mixture tested in mass-based units (µg/mL), whereas sorafenib is a purified compound evaluated on a molar basis (µM). Therefore, this comparison is intended to illustrate relative selectivity trends rather than to imply equivalence in pharmacological potency or clinical performance. Epidemiological and preclinical evidence similarly indicates that higher carotenoid levels are associated with reduced risks of several cancers, and that carotenoid-mediated antioxidant and signaling effects play significant roles in these protective phenomena [46]. Although the IC_50_ values of the *G. dulcis* extract are in the tens of µg/mL range, this level of potency is comparable to many crude plant extracts reported in HCC models and reflects the use of a complex phytochemical mixture rather than purified compounds. Importantly, these concentrations should not be interpreted as direct therapeutic doses, but rather as proof-of-concept evidence of biological activity at the cellular level. In vivo bioavailability of xanthophylls is influenced by absorption, metabolism, and tissue distribution, and plasma concentrations achievable through dietary intake are likely lower than those used in vitro.

It is also important to consider that the biological activity of the *G. dulcis* extract may arise from synergistic interactions among multiple xanthophylls and co-occurring phytochemicals, rather than from the action of individual compounds alone, which is a common feature of plant-derived mixtures. Besides xanthophylls, the extract may also contain minor levels of other bioactive constituents typical of *Garcinia* species, such as phenolic acids, flavonoids, and xanthones, which were not the primary focus of this targeted metabolomic analysis but could contribute additively to the observed biological effects. At the same time, it should be acknowledged that the use of only two hepatocellular carcinoma cell lines and one normal hepatocyte line represents a preliminary experimental model, and broader validation using additional HCC subtypes and in vivo systems will be necessary to confirm the generalizability of these findings. Therefore, the present findings should be viewed as supporting the mechanistic potential of the extract and its constituents, rather than implying immediate clinical applicability.

Collectively, these findings position *G. dulcis* xanthophylls as promising candidates for further mechanistic and translational evaluation in hepatocellular carcinoma. The predicted targeting of EGFR, BCL-2, and mTOR provides a mechanistic explanation for the observed antiproliferative effects of the Garcinia dulcis extract in HepG2 and Huh7 cells. Inhibition of EGFR and mTOR is expected to suppress proliferative and metabolic signaling, while modulation of BCL-2 directly promotes mitochondrial apoptosis, collectively leading to reduced cell viability. Thus, the convergence between in silico target prediction and experimental cytotoxicity supports a biologically coherent multi-target mechanism underlying the anti-HCC activity of the extract.

## 5. Conclusions

This study shows that *G. dulcis* pulp is a xanthophyll-rich matrix with strong anti-hepatocellular carcinoma potential, supported by integrated metabolomic, in silico, and in vitro evidence. Zeaxanthin and lutein dominated extracts displayed high predicted antineoplastic and pro-apoptotic activities, targeted key HCC pathways, and exhibited stable binding to EGFR, BCL-2, and mTOR in docking analyses. These computational findings were validated experimentally by selective cytotoxicity against HepG2 and Huh7 cells with a superior therapeutic window compared with sorafenib. Collectively, *G. dulcis* xanthophylls represent a promising multi-target natural candidate for functional food or nutraceutical development in hepatocellular carcinoma therapy.

## Figures and Tables

**Table 1:** Quantitative UHPLC-Based Profiling of Xanthophylls in *Garcinia dulcis* Pulp Extracted by MAE.

Compounds	Molecular Formula	Retention Time (min)	Yield GDP-MAE (mg/100 g)
Fucoxanthin	C_42_H_58_O_6_	11.042 ± 0.020	1.9 ± 0.2
Astaxanthin	C_40_H_52_O_4_	11.810 ± 0.050	1.3 ± 0.1
Zeaxanthin	C_40_H_56_O_2_	10.187 ± 0.051	29.8 ± 2.1
Lutein	C_40_H_56_O_2_	10.476 ± 0.054	21.6 ± 1.8
β-Carotene	C_40_H_56_	19.900 ± 0.200	3.4 ± 0.3
β-Cryptoxanthin	C_40_H_56_O	3.310 ± 0.010	6.1 ± 0.5
Canthaxanthin	C_40_H_52_O_2_	3.110 ± 0.020	1.2 ± 0.1

**Table 2:** In Silico Anticancer Activity, Toxicity, and Drug-Likeness Profiling of *Garcinia dulcis* Xanthophylls.

Compounds	Pa Score			Toxicity Model Computation Analysis		Drug-Likeness		
	Antineoplastic	Apoptosis Agonist	TP53 Expression Enhancer	Predicted LD50 (mg/kg)	Toxicity Class	Lipinski Rule	Pfizer Rule	GSK
Fucoxanthin	0.973	0.510	0.479	130	3	Accepted	Accepted	Rejected
Astaxanthin	0.907	0.919	0.905	1190	4	Accepted	Rejected	Rejected
Zeaxanthin	0.920	0.926	0.920	10	2	Rejected	Rejected	Rejected
Lutein	0.913	0.889	0.731	10	2	Accepted	Rejected	Rejected
β-Carotene	0.931	0.943	0.925	1510	4	Rejected	Rejected	Rejected
β-Cryptoxanthin	0.915	0.923	0.934	10	2	Rejected	Rejected	Rejected
Canthaxanthin	0.900	0.925	0.912	10,000	6	Rejected	Rejected	Rejected

**Table 3:** Molecular Docking Binding Affinities of *Garcinia dulcis* Xanthophylls Against Key Hepatocellular Carcinoma Targets (EGFR, BCL-2, and mTOR).

Compounds and Control as Ligands	EGFR (1M17)	BCL2 (2W3L)	MTOR (4JSV)
Doxorubicin (Control)	−9.8	−9.1	−10.9
Fucoxanthin	−8.9	−8.8	−10.5
Astaxanthin	−8.9	−9.0	−9.9
Zeaxanthin	−8.7	−8.8	−9.6
Lutein	−8.8	−8.9	−9.5
β-Carotene	−8.7	−8.9	−9.7
β-Cryptoxanthin	−8.6	−8.8	−9.5
Canthaxanthin	−8.7	−8.9	−9.6

**Table 4:** Cytotoxic Activity and Selectivity of *Garcinia dulcis* Pulp Extract and Sorafenib Against Hepatocellular Carcinoma and Normal Liver Cells.

Group	Type of Cell	IC_50_ (Mean ± SD)	Measurement	Selectivity Index (SI)
Extract *Garcinia dulcis*	HepG2	42.8 ± 3.6	µg/mL	4.27
	Huh7	58.4 ± 4.9	µg/mL	3.13
	THLE-2 (Normal)	182.6 ± 15.3	µg/mL	–
Sorafenib (Drug Control)	HepG2	6.2 ± 0.7	µM	2.39
	Huh7	7.9 ± 0.9	µM	1.87
	THLE-2 (Normal)	14.8 ± 1.4	µM	–

Values are expressed as mean ± SD (*n* = 3). IC_50_ values were calculated by nonlinear regression of MTT assay data. The selectivity index (SI) was defined as IC_50_ in normal THLE-2 cells divided by IC_50_ in cancer cells (HepG2 or Huh7), with a higher SI indicating greater tumor selectivity. One-way ANOVA with Tukey’s post hoc test showed significant differences among cell lines (F(2,6) = 128.4, *p* < 0.0001). Pairwise comparisons showed significant differences between HepG2 vs. THLE-2 (*p* < 0.0001), Huh7 vs. THLE-2 (*p* < 0.0001), and HepG2 vs. Huh7 (*p* = 0.012).

## Data Availability

The datasets used and/or analyzed during the current study are available from the corresponding authors on reasonable request. Further inquiries can be directed to the corresponding authors.
